# Transformational Change in maternity services in England: a longitudinal qualitative study of a national transformation programme ‘Early Adopter’

**DOI:** 10.1186/s12913-021-07375-3

**Published:** 2022-01-12

**Authors:** Beck Taylor, Alistair Hewison, Fiona Cross-Sudworth, Kevin Morrell

**Affiliations:** 1 Institute of Applied Health Research, University of Birmingham, Edgbaston, Birmingham, B15 2TT UK; 2 School of Nursing, University of Birmingham, Edgbaston, Birmingham, B15 2TT UK; 3 Cranfield School of Management, College Rd, Cranfield, Wharley End, Bedford, MK43 0AL UK

**Keywords:** National Health Service, Large-scale change, Health systems change, Health Care Reform / organization & administration, Health Policy

## Abstract

**Background:**

Large system transformation in health systems is designed to improve quality, outcomes and efficiency. Using empirical data from a longitudinal study of national policy-driven transformation of maternity services in England, we explore the utility of theory-based rules regarding ‘what works’ in large system transformation.

**Methods:**

A longitudinal, qualitative case study was undertaken in a large diverse urban setting involving multiple hospital trusts, local authorities and other key stakeholders. Data was gathered using interviews, focus groups, non-participant observation, and a review of key documents in three phases between 2017 and 2019. The transcripts of the individual and focus group interviews were analysed thematically, using a combined inductive and deductive approach drawing on simple rules for large system transformation derived from evidence synthesis and the findings are reported in this paper.

**Results:**

Alignment of transformation work with Best et al’s rules for ‘what works’ in large system transformation varied. Interactions between the rules were identified, indicating that the drivers of large system transformation are interdependent. Key challenges included the pace and scale of change that national policy required, complexity of the existing context, a lack of statutory status for the new ‘system’ limiting system leaders’ power and authority, and concurrent implementation of a new overarching system alongside multifaceted service change.

**Conclusions:**

Objectives and timescales of transformation policy and plans should be realistic, flexible, responsive to feedback, and account for context. Drivers of large system transformation appear to be interdependent and synergistic. Transformation is likely to be more challenging in recently established systems where the basis of authority is not yet clearly established.

## Background

The call for fundamental change in health systems is a familiar feature of health policy [1]. However, it has been estimated that between 50–70% of organisational change projects are unsuccessful [2, 3], and this is reflected in health care organisations [4]. While nomenclature varies, Large System Transformation (LST) [5], Large Scale Change [6], and Major System Transformation [7], broadly involve a process of change involving multiple stakeholders and organisations to achieve goals of improved quality, outcomes and efficiency. System-level change may also involve reconfiguration or integration *as a new system* (e.g. implementation of English Integrated Care Systems (ICSs) [1, 8],) or transformation *within an existing system* (e.g. 1990s Denver Health system redesign [9]). Greater understanding of this process is required if ambitious health policy aspirations are to be realised [10].

Health systems are dynamic, interdependent and unpredictable. Flexibility and responsiveness are required if transformation is to work [5, 11]. In England, the National Health Service (NHS) is a system serving the majority of the population, although in practice it is delivered in a fragmented way, by individual organisations and groups of organisations [12, 13]. There have been many attempts to integrate the NHS and related organisations [14]. The most recent was in 2016 when 44 ‘place based’ Sustainability and Transformation Partnerships (STP) were established in England. Each covered an area with populations ranging from 300,000 to 3 million people. The intention was to join NHS, local government and other organisations together, to improve health and services [15, 16]. STPs are changing once again, transitioning to more autonomous Integrated Care Systems (ICSs), with around half having already made this shift [17].

As part of the national move to systems approaches in healthcare, in 2016 a National Maternity Review, *Better Births*, recommended transforming maternity care in England [18]. Large-scale reorganisation of maternity services is not new: previous UK NHS programmes have included *Changing Childbirth* (1993), the *All Wales Normal Birth Pathway* (2003), and the *Scottish Keeping Childbirth Natural and Dynamic* (2007) [19–21]. However, in addition to pathway and care changes, *Better Births* recommended structural organisational changes, including the creation of new Local Maternity Systems (LMSs), coterminous with English STP/ICS boundaries, to increase the integration of maternity care services. This Large System Transformation (LST) therefore had two dimensions: 1) shifting *across* to a new, multi-provider Local Maternity System (LMS), and 2) reforming care *within* the new system.

We evaluated one of eight ‘Early Adopter’ LMSs [22], which were funded by government to implement transformation within two years. The evaluation was underpinned by the ‘simple rules’ for LST developed from a realist synthesis of relevant evidence [5]. This provided an opportunity to contribute to the evidence base by examining the utility of the rules, and exploring the implementation of a national policy-driven cross-provider LST as it unfolded, within the context of new and emerging health system structures in England. We explore whether and why the LST approach aligned with ‘simple rules’ for LST. We also critique of the rules as a framework to support and evaluate policy-driven LST in complex health systems.

## Method

### Study design

A longitudinal, qualitative case study was undertaken in three phases: Phase 1 January to December 2017; Phase 2 January to December 2018 in year 2; Phase 3 January to May 2019.

### Setting

The study was of a Local Maternity System (LMS) in England, serving a diverse, predominantly urban population of over one million, with nearly 20,000 births each year. Stakeholder organisations included two NHS ‘Trusts’ (responsible for services in four hospitals), two local authorities, and other local health and social care organisations, and charities. The LMS successfully applied to be an ‘Early Adopter’ of the national *Better Births* policy, and received funding to expedite transformation between 2016 and 2018 across five areas: personalised care, continuity of carer, postnatal and perinatal mental health care, electronic patient records, and novel payment methods.

### The Evaluation Framework

Founded on a realist synthesis of the international evidence [5], we used the ‘simple rules’ framework for success in LST to inform the evaluation. This was originally developed by Allan Best and colleagues but we also drew on other work where these rules have been applied and further refined, including integrated care initiatives [23], stroke services [24], and primary care [25]. While we were unable to locate examples of the rules being applied in maternity services, they have been used as an evaluation framework in similarly complex LST programmes in UK healthcare. These involved integration and shifting of services between large, mature organisations, including divestment in previous ways of working [24, 25]. As a realist synthesis, the context in which LST occurs was a central focus of the Best rule development. The rules are:

1. Blend designated and distributed leadership
2. Establish feedback loops
3. Attend to history
4. Engage physicians
5. Involve patients and families

### Participants, sampling, recruitment and data collection

We had three main data sources: interviews and focus groups with staff, women and families; non-participant observation of meetings and events; and relevant documents (see Table 1). While all sources of data were analysed, we only draw on the data from the individual and focus group interviews as exemplars in this paper, as they offer the most rich and illustrative evidence to support the analysis.

**Table 1:** Data collected

Data type	Phase 1	Phase 2	Phase 3	TOTAL
Interviews	18	45	20	**83**
Focus groups (see Table 2)	2	9	16	**27 (162 participants)**
Documents	47	80	48	**175**
Meeting observations	12	14	14	**40**
**TOTAL**	**79**	**148**	**98**	**325**

#### Interviews and focus groups

The approach to data collection was slightly different in each phase, reflecting the data required at each stage, and the progress and scale of the LST. In phase 1 the LST involved interviews with 18 senior leaders from the organisations involved (including a women’s representative), and one senior midwife focus group at each of the two main providers. In Phase 2 the change had progressed, and a wider range of data was gathered (focus groups with frontline staff and women’s representatives involved in the programme), and senior midwives were interviewed at this point to gather more in-depth individual data regarding participants’ specific roles in the programme. In Phase 3 additional focus groups were held with women and obstetricians not directly involved in the programme, and senior midwife accounts were gathered by focus group to gather perspectives of the programme as a whole.

Interview participants were identified purposively, based on their role in the LST and health system, and contacted directly by researchers. Participants included the programme team, senior clinicians (obstetricians, midwives, mental health professionals), and NHS and Local Authority leaders/managers. Twenty nine interviews were undertaken with senior clinicians and 54 with other leaders and managers. Interviews were face-to-face in workplaces, or – rarely - by telephone when scheduling challenges meant this was not possible.

Twenty seven focus groups were conducted with 162 participants: 115 midwives, 22 Midwifery Support Workers/Midwifery Assistants, 14 student midwives, and 11 obstetricians. For the senior midwife focus groups, participants were purposively sampled by role and organisation with key senior staff from each organisation represented, and focus groups held separately at each site. Frontline midwifery staff focus groups were held in convenient locations within workplaces during the working day, in purposively sampled hospital and community maternity sites, to ensure midwives, MSWs and students from all organisations and settings were represented. Separate focus groups were held for staff from different organisations and settings, with staff of all grades (midwife, MSW, student) ‘on shift’ in the community or hospital site on the day they were invited to take part. One obstetric consultant focus group was conducted at each organisation, and invitations circulated by a senior obstetrician in advance.

Women participants were sampled differently in each phase, reflecting their increasing involvement in the LST over time. In phase 1, one key informant was identified and interviewed. In phase 2 we identified women directly involved in the LST to take part in one focus group. In phase 3 we worked with local organisations, holding one purposively sampled focus group with women directly involved in the LST. To explore the awareness and impact of the LST among the wider population we held three further focus groups with women who were current or recent service users and not involved in the LST, each in a different location and community, selected to maximise diversity of participants. The locations for women’s focus groups were chosen by participants or their representatives (e.g. at home, a café, a community centre). Fourteen women directly involved in the LST, or who were recent service users also participated in five focus groups (see Table 2).

**Table 2:** Focus groups

Participants	Phase 1	Phase 2	Phase 3	TOTAL
Midwives/Midwifery Support Workers Midwifery Assistants /student midwives	0	8	7	**15**
Senior Midwives	2	0	3	**5**
Obstetricians	0	0	2	**2**
Women	0	1	4	**5**
**TOTAL**	**2**	**9**	**16**	**27**

Specific topic guides, informed by the Best rules, were developed for the individual and focus group interviews. Interviews and focus groups were conducted and facilitated by [authors x, y, and z] who are clinical researchers with backgrounds in public health medicine, nursing and midwifery respectively. Interviews and focus groups lasted between thirty and ninety minutes, and interviewing, moderation and facilitation duties were distributed equally among researchers. Written informed consent was obtained from participants. All interviews were digitally recorded and transcribed verbatim for analysis, supplemented by contemporaneous notes taken by researchers during interviews and focus groups. In total we recorded over 100 h of such data.

#### Non-participant observation

Researchers observed key meetings during the data collection periods, including Board, senior team meetings, workstream meetings, engagement and involvement activities. Access to meetings was negotiated with the programme leaders and informed verbal consent was obtained. Notes were taken during the meeting using a standard template recording key aspects of discussion and context.

#### Document Review

Documents were identified by interview and focus group participants, and by searching policy and organisational websites. Documents included service reports, papers from the Programme Board, Workstreams and subgroups (minutes of meetings, strategy documents), and external communication (e.g. newsletters). Meetings and events observed included those of the transformation programme Board, Workstreams, Task and Finish groups, ‘Maternity Voices Partnership’, ‘Stakeholder Council’, and staff engagement events.

### Analysis

Transcripts from interviews and focus groups, observation notes, and documents were managed using NVivo**©** 11 and analysed thematically using the framework method, a systematic approach to managing and interpreting data, particularly suitable for large, complex datasets [26]. In each phase, three exemplar transcripts were coded independently by the research team, applying an initial coding index which reflected the research questions, including the five rules, key aspects of the Better Births policy, with space for additional codes derived inductively from the data. Researchers met to discuss assignment and interpretation of codes, and develop an analytical framework for the remainder of the data. This method was used in each phase of the study, and separate framework matrices were constructed in each phase. In each phase, once satisfied that data saturation had been reached [27], analytic summaries of the main findings were written for the Best rules and other key themes. These were integrated in each evaluation phase, to provide an emergent account of change over the course of the LST. Themes which did not address the Best et al. framework directly are not presented in this paper, but all were reviewed to aid interpretation of the alignment of the LST with Best’s rules. The longitudinal analysis [28] centred on the researchers’ interpretation of change over time in terms of: 1) alignment with each of the Best rules, and 2) the structure, context and progress of the LST.

### Ethics and governance

University of Birmingham Ethics Committee approved the work reference number ERN_16-1452. Research Governance teams at the participating NHS trusts provided approval to conduct the work. All methods were performed in accordance with the relevant guidelines and regulations.

## Results

### Participants and data

Three hundred and twenty five data items were collected (see Tables 1 and 2).

### Transformation programme structure

**Fig. 1:**
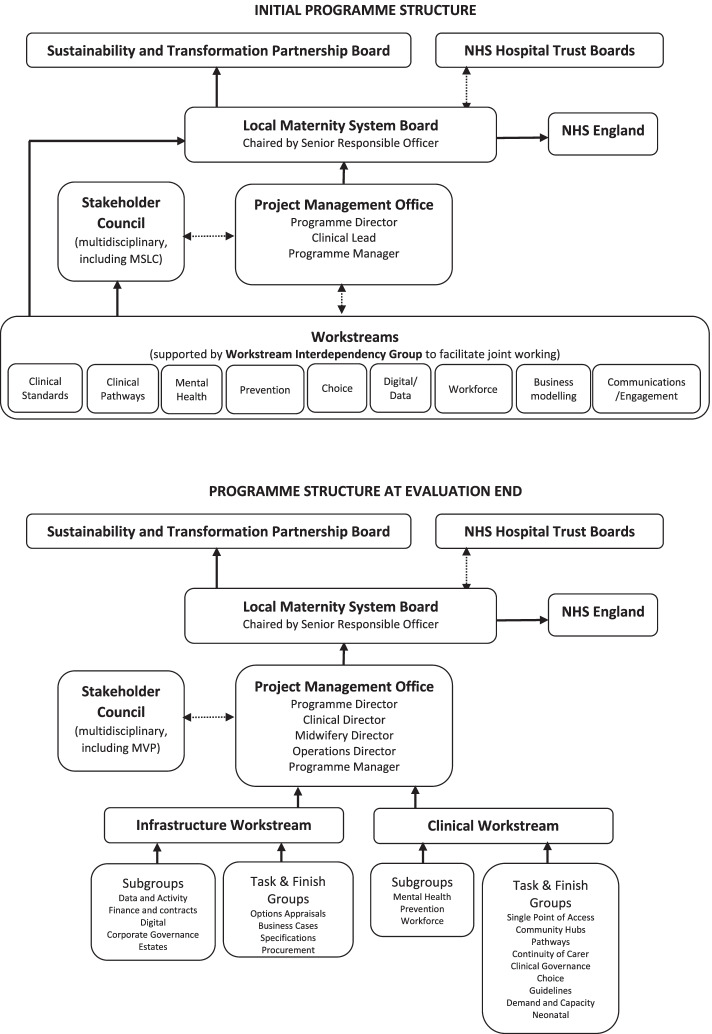
Structure of the maternity transformation programme

The programme organisation evolved over time, and the structure at the start and end of the study is illustrated in Fig. 1. Throughout, it was overseen by a Programme Board which reported to the local Sustainability and Transformation Partnership Board. Initially, nine workstreams were established, led by clinical and non-clinical leaders drawn from across the NHS and local authorities with appropriate expertise and seniority, with a ‘Workstream Interdependency Group’ to integrate the programme. The workstreams reflected the national policy team’s workstream structure. This was subsequently consolidated to two workstreams led by directors in the programme team, with a number of working groups and task and finish groups led by programme directors and other leaders as with the initial workstreams. A ‘Stakeholder Council’ provided scrutiny and input, with membership drawn from public sector and third sector stakeholders, including the Maternity Services Liaison Committee and later the Maternity Voices Partnership (discussed later).

### Alignment with simple rules for LST

Below we present the main findings in sections that correspond to the rules for LST [5].

#### Blend designated and distributed leadership

At the start of the LST, leadership was the responsibility of senior staff drawn from one of the main provider organisations, including a full-time Programme Director. In 2018 this team was disbanded and a new, larger leadership team recruited including Medical, Midwifery, Operations and Programme Directors. This interruption in leadership was reported to have hampered progress.

*The leadership team [for the programme] has changed completely and I think for me although I did really enjoy [the staff engagement event] and it was a good opportunity it felt like a conversation we’d had twelve months previously. Clinical Leader 1, Phase 2*

Other leadership roles were distributed across discrete areas of work. A lack of balance in the leadership representation across the two main provider organisations led to tension and disengagement initially and the programme was restructured to address this. Subsequently, designated leaders (Medical, Midwifery, Operations and Programme Directors) were recruited, predominantly from outside the participating organisations, and other roles were redistributed more widely.

*I think it’s helped by having people from outside of the organisations but also then the difficulty for them is trying to understand the organisations whilst also doing this kind of transformational role. Obstetrician 1, Phase 2*

The team found that having nine workstreams involved many meetings in different locations, and attendance was variable, communication disjointed, and ‘silo’ working was evident. Consolidation into two workstreams, led by two senior leaders, provided a clearer focus and increased participation, though it was still challenging bringing stakeholders together.

*[The Workstream Interdependency Group] never worked properly and actually having [the new structure] actually works much better…things have been done in a much clearer way so you know where things are going. Clinical Leader 2, Phase 2*

The national policy and ‘Early Adopter’ funding resourced system-level leaders, although their limited involvement in operational leadership meant that LST was seen as peripheral to core business. National policy did not specify leadership structures, and participants suggested that local appetite, vision and funding for system-level roles in the long term were uncertain.

*It [future system leadership] can work ultimately… money would come from top slicing organisations and do the organisations really want that or need that, probably not actually… I think that if I was a provider would I want, I don’t know 100,000 top sliced off to pay the [System Leaders] to bring things together? Programme Leader 1, Phase 3*

Distributed leaders questioned aspects of the national policy, and struggled to articulate the evolving vision of ‘the system’ which was not defined in statutory terms in policy or practice.

*So I think for me what’s been lacking here is the strategic direction of the LMS in the longer term and it’s hard to be working without that strategic direction, what are we trying to achieve? Clinical Leader 2, Phase 3*

Such concerns were exacerbated by concurrent, overlapping national initiatives and the expectation that they would be implemented in the new system.

*We've got the recommendations from Better Births. Now we're being asked about Saving Babies Lives [a separate initiative]. How does that fit in? I think it took a little while to piece it all together. Programme Leader 3, Phase 3*

Leadership culture, skills and practice were reported to be organisation-, rather than system-focused. A shift towards an integration and ‘system-mindset’ was reported to be challenging, for example:

*Over the last 10-15 years, [NHS] managers and clinicians have been groomed to think 'my organisation' and 'sovereignty of my organisation' and not 'what can I learn by working collaboratively and how do I bring my colleagues into this?' Senior Midwives Focus Group, Phase 3*

Capacity and funding to undertake transformation work alongside existing responsibilities were reported to be limited.

*I've been asked to do a whole load on the [topic area]…There is no time made for me to do it. I'm not paid to do it at all. When the meetings come and I've already got a scan list or something, I just think, 'I've got my scan list. I can't go.' Obstetrician Focus Group 2, Phase 3*

Receptivity and capacity of distributed leadership were also felt to be limited by constant change.

*I guess we were trying to make change against a backdrop of change. We'd got [the hospitals] merging. We'd got the [commissioners] merging…Obviously, we'd got the change with the Project Team as well. Programme Leader 3, Phase 3*

Programme leaders had limited power to challenge resistance and align leadership in separate statutory organisations that were not formally accountable to the system, which itself was at no point clearly defined. There was also a perception that LST was not a priority for some.

*Even if we had perfect engagement and everyone lined up, it would still be extremely difficult to do within two sovereign organisations. The fact that we’ve got two sovereign organisations and there’s a whole host of people that don’t want to do this, that’s going to cause huge problems in the future. Programme Leader 6, Phase 3*

The respondents did not feel existing structures facilitated LST and that a shift of power and authority from the trusts to the LMS was needed.

#### Establish feedback loops

The complexity and scale of the LST, and concurrent local and national quality improvement initiatives, created challenges in determining what to measure, and how to gather and feed back information. While specific performance indicators were set by the national policy team, a much wider range of measures had to be developed to monitor the progress of LST locally. There was also overlap in data requirements for measuring core LST objectives, and national and local targets from other quality improvement initiatives. For example:

*I think what hasn’t worked well is the level of information required… it’s required by different levels of the organisation in different formats and different ways…And actually the information is usually the same, but the templates and the way they want it is usually different. Programme Manager 1, Phase 3*

Frontline staff and patients were not actively involved in agreeing the measures, in part because many had already been set centrally, e.g. continuity of carer targets. This was compounded when additional targets were set by local strategic system leaders with limited knowledge of maternity services, such as a home birth target of 5%. Recent implementation of a dedicated home birth service had achieved a substantial increase in home births, though the rate of change in home births meant that achieving 5% was not considered feasible within the expected timeframe.

*One of the things they said was that they would get to a five per cent home birth rate… they're never going to get to five per cent … So I think that was unrealistic and we haven't been held to account for that. Programme Leader 8, Phase 3*

A number of interviewees identified gaps in reporting processes and lack of accountability, particularly with regard to the impact of involvement and engagement (a theme we return to in the discussion). Attempts to integrate data across organisations were felt to be hindered by gaps in information sharing. This was reported to arise from fears within ‘sovereign’ organisations that data sharing would breach information governance policies.

External reporting was focused on sharing ‘good news’. There was less sharing of challenges (e.g. delays), thereby reducing opportunities for scrutiny, challenge, and learning. Senior leaders also described the challenges involved in communicating with multiple organisations within a ‘system’ that was still developing. Stakeholders wanted regular feedback, even when there was ‘no news’, about the impact of their input. Communication gaps resulted in disengagement, anxiety, and a sense that the change would never happen.

*F: I think if there was a continuous thread from the start and that we were involved with giving our opinions, we would have been involved then in hearing back…*

*F: It's almost dissolved, hasn't it? For us, there was a big [transformation programme] thing going on and now it's just dwindled. I just think, 'That's not happening.'*

*Community midwife focus group 1, trust 2, Phase 3*

In addition some respondents felt changes in the targets set to measure service performance, made at a National level during the LST, were unfair and created the potential for misreporting. In particular, participants reported how the definitions of continuity of care models were refined during the course of the LST, and how the targets for the proportion of women booked onto these models was increased and changed to focus on booking disadvantaged and minority women. Participants described how continuity targets would be difficult to achieve due to challenges in workforce capacity and willingness to work in this way.

*There’s a bit of an anxiety about the goalposts being stretched, so actually just as we’re getting our heads around the percentages [of women booked onto continuity of care pathways] that we’re expected to deliver, we know that that’s going to increase next year and actually, just seeing it’s unobtainable, makes you wonder whether people will fudge results just to kind of access money. Trust 1 senior midwives’ focus group, Phase 3*

Targets were set for some aspects of the LST (e.g. aforementioned national ‘continuity of care’ targets), although this shifted the focus of transformation efforts onto these areas, a risk acknowledged in Best et al’s work. Initially, measurement was difficult, data quality varied, and manual audit was sometimes required. However a new electronic patient record was introduced over a period of two years which enabled more accurate measurement of activity.

*We know the data quality here is really poor and when you start to look at the processes... it’s not that surprising really. We’re so paper-based and so reliant on people sending things in. Non-clinical Leader 7, Phase 2*

Measurement was delayed by limited availability of analysts and a reluctance to share data. In addition the partner organisations prioritised operational data management over that required for the LST. Some data was disregarded (e.g. caesarean section rates - a key performance indicator - because caesarean section was defined differently at national, regional and trust level). The data was reported to have greater integrity as the work progressed.

*I think we’re at that stage with the dashboard where people don’t believe it yet because they’re not sure about the data and some of the data is definitely not correct, but the data’s not correct because they’ve not submitted the data! Manager 1, Phase 3*

Two different digital sharing platforms were trialled to facilitate feedback loops within the programme, but staff had limited capacity, skills and commitment to use them, exacerbated by management discontinuity.

*I hadn’t realised we would have to put so much historic stuff in [to the new digital project management platform] to make it generate information for us…because the whole team went, I had no intelligence. Manager 1, Phase 3*

#### Attend to history

Participants frequently described local and national history, including recent mergers, and competition and the unequal power balance between the provider organisations now required to work as ‘one system’.

*I think you need to know the history, you definitely need to know the history of the two trusts because it’s going to affect how trusts see one another. Because they almost see it as, if you think for [Trust 1 Site 2] they’ve gone through two [mergers] already and this is like a third… Clinical Leader 9, Phase 2*

While participants naturally gave accounts of past events, they seemed less likely to openly engage with history in the sense Best et al. [5] recommend - of acknowledging the role of path-dependence, and the importance of context in understanding the rationale for change. Instead, the need to break with the past was identified along with an emphasis on pragmatism.

*I: What role does history play in what’s happening locally within the … Transformation?*

*IV: I would say none because history is past. Does it give you an insight into the conversations? Probably. I prefer to take things at face value and start from where we are at, set our goals going forward and then just work from here onwards. I think you can spend a lot of time analysing the ‘whys’ and ‘wherefores’ and the background and actually, it’s probably a wasted effort.*

*Programme Leader 1, Phase 2*

Discontinuity in staffing, and changes in leadership roles, appeared to restrict participants’ focus to immediate demands and perhaps led to a reluctance to speculate on the past.

*It’s very difficult because I didn’t start with this project…So I can’t reflect on what the good, the bad and the ugly was really because I don’t think that’s fair for me to do that.*

*Programme Leader 8, Phase 2*

Accompanying this pragmatism, participants were unwilling to criticise previous strategic choices, suggesting an inability to learn from history:

*I think it was clear that we had gone down the wrong way but that’s really easy for me as a new person coming in because I had no emotional attachment to the work.*

*Programme Leader 3, Phase 2*

Other factors contributed to what we came to see as a kind of revisionism or ‘editing out’ of history. Many staff were still adjusting to pressures resulting from a recent wave of hospital mergers. The new system was also expected to encompass key organisations and communities that in the past had not been formally linked. Stakeholders did not always feel able to acknowledge or learn from past ‘failures’ because local and national leaders expected policy to be delivered and were only interested in ‘success’. A key document designed to encourage continuity of midwifery care in the 1990s - *Changing Childbirth* [29] - was deemed unrealistic because of the reported impact on the workforce.

*F: And I think to some extent as well we’ve sort of been asked to forget what’s happened before because this is new, this is different and not be affected by our memories of Changing Childbirth, dare I say it, but it isn’t, you can’t forget it because…*

*F: Yes because if you say, “Well we’ve tried this before,” you are seen as a ‘negative Nelly’ almost, you know, that you won’t try new ideas and that’s not true, you know, you are not allowed to say that.*

*Senior Midwife Focus Group Trust 2, Phase 3*

#### Engage physicians

As well as recognising the need to engage physicians, participants emphasised the role of other stakeholders. Midwives were seen to be particularly important. Multispecialty health providers, local authorities, primary care, mental health, children’s services, and third sector organisations were also involved. Initial engagement was predominantly via stakeholder events, with some newsletters, social media, clinical site visits and meetings.

The Programme Team planned and delivered most of these events. A communications and engagement lead was appointed in year 2, which increased the amount and quality of activity.

*We’ve had more in the last three months through [dedicated communications and engagement lead] than we’ve had in all the time I’ve worked in [the programme], …, and I guess it comes back down to the resource again. Manager 3, Phase 3*

Assumptions that distributed leaders would see engagement as their responsibility were not always met.

*I don’t think it’s [engagement] been as good for [my service], I did say this in the meeting recently and somebody said to me, ‘But actually you’re the… person, isn’t that part of your responsibility?’ and I thought ‘Actually, yes.’ Clinical Leader 11, Phase 3*

Leaders reported significant engagement, whereas stakeholders suggested that it was not sufficient or meaningful. This was exacerbated by the programme team not being well-known to frontline staff.

*You feel like decisions are made before you even get to know and it’s usually all happening before you’re told.*

*Hospital midwives’ focus group trust 1 Phase 3*

This resulted in some frustration among the leadership team:

*Some of the comments were that we don’t get to hear anything on the front line but what more can we do, so I said “Look we’ve got the newsletter, would you read a newsletter?”… “Well we don’t have time to read a newsletter.” …we have asked on many occasions where can we come to your meeting that you have with your community teams… and it’s still to happen. Manager 3, Phase 3*

Leaders described difficulties in engaging many busy, diverse stakeholders across organisations and sites, identifying particular problems at the middle management level.

*If you spoke to the boards of the two organisations…, I think they would say we were absolutely on the same page. …It seems to get stuck in that middle management layer. …it's almost like you've got to explain yourself again. We have even been asked, 'What is [the programme]? What's the rationale? What's the legal basis?' Programme Leader 3, Phase 3*

There were also differences in perceptions of what counted as ‘engagement’. At times this simply meant communication (telling), rather than genuinely involving people.

*If you engage with people too soon, they complain that you haven’t got the answers for them. If you then engage with them later on when you’ve got all the answers, they complain that you haven’t involved them in designing it in the first place. Programme Leader 6, Phase 3*

Levels of engagement were also influenced by policy imperatives and targets.

*I think the game-changer was Saving Babies’ Lives [new national targets] because suddenly, “Oh, smoking in pregnancy is important, and smoking in pregnancy isn’t something that just Public Health do.” Non-clinical Leader 11, Phase 3*

Some clinicians received payment for their input, which appeared to encourage engagement, whereas others reported not having time for meaningful involvement. While the underpinning *Better Births* policy recommendations appear clear, translating this into a meaningful, tangible local vision and engaging stakeholders in such a complex, emergent change with multiple uncertainties, and demanding timescales, was challenging. System-wide change at this scale was unprecedented, and often the externally-set policy priorities did not align closely with staff priorities for local change, for example *Better Births* does not mention health inequalities in maternity care.

*It’s hard because I don’t think we are entirely clear on the vision, I think it’s hard for staff to see why we are doing it really. Clinical Leader 2, Phase 3*

Maintaining momentum was also difficult, and ambitious goals were not achieved as planned or ‘on time’. Some respondents believed change was unlikely.

*It was supposed to start in April or May this year and we’re nowhere near ready for that. Which is fine and that’s okay that we’re going to get it right before we start of course. But it seems like this vision in the future that I’m not sure we maybe lose a bit of momentum when it goes on a little bit don’t you, that you’re not sure it’s going to happen. Manager 4, Phase 2*

Stakeholder receptivity was greater if the changes were seen to be relevant, welcome, imminent, and focussed on specific targets, and where change had already occurred. Respondents were less receptive in situations where they had negative experience of past change efforts because it was enforced, or where ‘change fatigue’ had set in.

*I think it's not really tangible yet for a lot of people. There is all this work going on in the background. Once it's hitting me in the face, I need to know more about it then, I think. Obstetrician focus group, Phase 2*

#### Involve patients and families

While a commitment to involve patients and families was expressed, activity was inhibited by uncertainty regarding resources, and strategy. Consequently the mechanisms for supporting system-level involvement were unclear.

*We thought, in our naivety that there was a little army of people who were going to ensure that all of the stakeholders were engaged … it was never there apparently, it was always going to be too expensive. Clinical Leader 12, phase 2*

Patient involvement was a national priority identified in *Better Births* and was reflected in the establishment of local ‘Maternity Voices Partnerships’ (MVPs), superseding previous Maternity Services Liaison Committees (MSLCs). However, MVP policy was announced some time after *Better Births* was launched, and MVPs were not active until a year into the programme. The local MSLC was retired, and a new model was commissioned by the local Clinical Commissioning Group (CCG). a third sector provider was contracted to manage the MVP. Participants described this approach as different to other English maternity transformation programmes, and how it was taken in order to strengthen links to the local community. The MVP included a lay Chair, Vice Chair, and manager, accountable to the CCG, but responsible for supporting the maternity LST and wider involvement activities for maternity, by undertaking a number of activities involving local families (e.g. meetings, surveys, focus groups). The local decision to commission the service from an external agency, resulted in a delay in establishment of the MVP. The LST was at an advanced stage before the MVP sought the views and comments of women.

*Well I guess that would be my own reflection as well is that we should have kick-started this whole thing with the MVP from the outset. Manager 2, Phase 2*

Later in the programme there were examples of effective involvement of the MVP.

*I think with the [digital] stuff we have shaped a lot because of the [MVP] input because when we first had the snapshots of what the portal might look like and what it will capture, how easy is it for women to access, especially with the language barriers and things like that, I think they’ve helped us with that a lot and it’s been changed a lot. Manager 3, Phase 3*

Involvement increased over time, though many still wanted more varied and frequent activities.

*I think probably more patient engagement [was needed]. Even for the group of women who are currently pregnant and accessing mainstream services, I think we should be doing lots more. Clinical Leader 13, Phase 2*

Leaders concurred that more involvement was desirable, however there was a gap between involvement principles and practice. Limitations identified included the rapid pace of change, leaders’ limited knowledge of and skills in engagement, and a reluctance to involve people early.

*We should have had service users, you know, co-designing with us and I’m not sure that other clinicians within the group thought that that’s what service user engagement meant… women weren’t really with us, you know, throughout the whole journey and I think people were irritated at the thought that it’s not the right time to bring them in yet. Clinical Leader 14, Phase 2*

The way involvement culture and practice had worked in the past influenced the approach, and commissioning a community provider was regarded as a positive move away from past structures.

*… some of communities that we'd been trying to reach we are reaching now where we weren't before [using previous local involvement structures], the Chair is a lady from a diverse community as is the Vice Chair which is really good. Programme Leader 8, Phase 3.*

Some women reported that the approach addressed barriers to involvement.

*F: So she [MVP worker] found out what's going on locally for mums to access, be that, you know, a nursery, like stay and play sessions and stuff. So she went round and she did that, footwork to recruit the women. And I think on the last one it was really picking up momentum, yeah. Women’s representative focus group 1, Phase 3*

Maternity-specific barriers to participation were reported. For example, pregnancy is a ‘temporary’ condition, and women are often busy with work and family.

*How can you get women who really do need [the transformation] to be shaped for them and who are women who work jobs where they’re not going to be able to come, have children to look after, or elderly relatives, or not necessarily able to leave the house. Women’s representative focus group, Phase 2*

Participants reported it was important to provide support for transport, financial incentives, and a suitable venue. Financial incentives varied across the programme.

*I think they should be paid, even if it's £10 an hour. That's an hour there and travelling and any feedback. It's not massive but just for the inconvenience of it really. Manager 5, Phase 3*

The scale of the system, the LST, partner organisations, and an extremely diverse community meant that achieving genuine involvement was complex and challenging. The MVP was also required to involve patients in both the LST and ‘business as usual’ improvement.

*It's almost two different roles [for the MVP]…They're not mutually incompatible or distinct but they are different. Programme Leader 3, phase 3*

The new MVP model was evolving, and early on there were some mismatches in stakeholder expectations, for example which women and communities the MVP was expected to involve, or what role different NHS staff (communications, clinical, management) would take in leading steering involvement. The LST team subsequently worked with the CCG and MVP to refine the contract to address this.

*What it came down to in the end is their [MVP’s] understanding of what they needed to contribute versus ours, building that relationship, we need to work together and I get that now… I think it’s a two way thing and I think from our perspective I think we just needed to work much more closely. Manager 3, Phase 3*

Further barriers to patient-centred involvement work, were a mismatch between local women’s priorities, and the national policy focus.

*I think the priorities are all skewed and if they’d started from the bottom up model rather than ‘these are our priorities and we’re trying to filter it all down’. Women’s representative focus group 1, Phase 3*

Leaders felt that it was important to manage expectations about the level of involvement the LST would achieve.

*I think we’ve had a great conversation with people about what they would like it to be, but we’ve got to be a bit careful that we don’t raise too much expectations Programme Leader 6, Phase 2*

Women’s representatives reported a desire for more support, feedback and recognition (see also rule 2, ‘feedback loops’).

*I’d given a lot of myself … and to not really hear anything back... Women’s representative focus group, Phase 2*

Involvement was more successful when it happened early, in discrete projects, which women valued and understood, e.g. the online patient portal. Although at the end of the evaluation, women still reported information gaps.

*I’d like to know just what it [the change programme] is, what are the objectives, what have they achieved so far, what changes have been made, where we are going in the future? Women’s representative focus group 1, Phase 3*

### Summary: interactions between the five ‘simple rules’

During the course of our analysis, we identified a number of interactions between the simple rules, where the alignment with one rule might trigger alignment with others. The dynamic and interdependent nature of the facilitators of LST is presented in Table 3. The issues involved are examined further in the discussion.

**Table 3:** Interactions between Best’s simple rules for Large System Transformation

	INFLUENCED				
INFLUENCER	Blend designated/ distributed leadership	Feedback loops	Attend to history	Engage stakeholders	Involve patients
**Blending designated/distributed leadership influences other rules by…**	–	Implicitly/explicitly requiring distributed leaders to disseminate and gather feedback in their part of the system, but leaders must understand that this is part of their role	Providing a blend of leaders with experiential knowledge, who know and can share history, though this does not guarantee that they will actively engage with it	Providing resource/expertise for engagement. Dedicated leaders provide recognisable ‘figureheads’ for change programme, driving strategy, though separate leadership team risks change being seen as ‘someone else’s business’, reducing engagement. Distributed leaders trusted insiders, can engage with their staff, increases capacity for engagement, opportunistic/ naturalistic. Leaders must have the knowledge, skills, credibility and capacity to engage effectively.	Providing a wider pool of experience, knowledge and capacity on which to draw. Designated leaders can set agenda and strategy for involvement, though need to ensure not seen as the sole leads for involvement. Distributed leaders may understand patients better, select more appropriate approaches, able to involve opportunistically. Leaders require knowledge, skills and capacity to involve patients effectively.
**Feedback Loops influence other rules by…**	Informing leaders how the current approach to leadership is working, and whether changes are indicated	–	Judicious quant and qual measurement of baseline and progress, providing an account of history on which to draw later	Enabling quant/qual feedback to be gathered from and shared with stakeholders to maintain momentum, evidence ‘you said, we did’ and encourage continued engagement	Enabling quant/qual feedback to be gathered from and shared with patients to inform involvement activities, maintain momentum, evidence ‘you said, we did’ and encourage continued involvement
**Attending to History influences other rules by…**	Enabling leaders to apply learning from past change, and to ensure sensitivity to political/ organisational issues.	Enabling leaders to learn from previous approaches to capturing and using measures.	–	Enabling leaders to learn from previous approaches to engagement. History can be actively discussed with stakeholders to engage and collectively deliver change.	Enabling leaders to learn from previous approaches to involvement. History can be actively discussed with patients to inform involvement and collective decision making.
**Engaging Stakeholders influences other rules by…**	Encouraging stakeholders to take on distributed leadership roles, and identify potential designated leaders	Enabling more appropriate and complete identification and gathering of measures to inform the change, and effective approaches to sharing	Enabling gathering different stakeholder accounts of ‘history’ to build complete picture	–	Drawing on stakeholder knowledge of approaches to reach and involve patients. Stakeholders may have more opportunity to reach patients in their routine practice. Can advise on the approach and lead/participate in patient involvement work.
**Involving patients influences other rules by…**	Encouraging patients to take on distributed leadership roles, e.g. chair involvement groups (ladder of participation).	Enabling measurement of baseline and progress from a patient perspective, and identify measures which are most important to patients.	Enabling gathering patients’ accounts of ‘history’ to build complete picture	Evidencing importance of the change to people system cares for. This may encourage stakeholder engagement in the change.	–

#### Additional themes

While only the core themes relating to the Best rules are reported in this paper, our combined deductive and inductive approach to analysis, and interrogation of additional (not reported) themes alongside the five Best rules, enabled us to explore ‘beyond the rules’. The other themes included detailed information about a range of issues which although related to the LST did not inform the analysis in the context of the Best rules. For example there was a lot of discussion of local innovations such as maternity triage which, although an interesting development, was an element of the transformation rather than being central to it. Similarly the development of the Electronic Patient Record was a distinct theme, however only the elements of it relating to the simple rules are included in this paper. A full list of the themes can be found in Table 4.

**Table 4:** Thematic structure^a^

Best rules	Blend designated and distributed leadership Establish feedback loops Attend to history Engage physicians Involve patients and families
Maternity transformation policy elements	Choice and personalisation Continuity Safer care Perinatal mental health Postnatal Neonatal care Multiprofessional working Electronic patient record Cross boundary working Payments, personalised maternity care budgets
The transformation programme	Structure and organisation Funding and resources Targets and vision Local innovations Support and sharing learning Capacity and time to transform Use of theory and evidence Approach to health inequalities Interdependencies and uncertainty Sustainability

^a^Includes themes relating to the Best rules, and additional themes from the wider evaluation, which are not reported in this paper

## Discussion

Over almost three years, the structure and approach of the LST was refined as circumstances changed, and learning about ‘what worked’ occurred. Alignment with the rules for LST varied. There was a tension between leading the programme centrally/separately to deliver change rapidly, and embedding distributed leadership and engaging stakeholders throughout the system [30]. Engagement beyond health services was difficult, an issue evident in other place-based transformation programmes [31]. Our findings offer some specific insights into LST where it requires the creation of a new system, and is driven by top down national policy.

### Systems, and change within systems

The dual nature of the LST in English maternity service transformation required 1) the creation of new Local Maternity Systems, and 2) concurrent development and implementation of changes in these systems. Our findings concur with Best et al. [5], in that we identified the significant challenges involved in implementing change into a system which is not established or recognisable to stakeholders, and for which there is no central accountability. Work continues to forge a new shared identity for a system whose boundaries were defined elsewhere, assumed to be meaningful and necessary, and where historical, organisational, geographical, and political differences were not accounted for in the policy directives [16]. While the launch of STPs following the 2014 *Five Year Forward View* [32] might be assumed to have started the process establishing the identity of the new coterminous Local Maternity Systems in England, we found that this was not the case in practice, and that the STP was still evolving. Better Births reinforced the need to work as a system further, in its recommendations for increased cross-boundary clinical working to improve care and safety. Despite this, stakeholders were generally organisation- rather than system-focused, arguably the legacy of a long history of NHS provider competition and quasi-market structures in the NHS [16]. Building trust incrementally in new systems is required, and is reliant on effective feedback loops to provide information about successes and setbacks [33]. Our findings suggest that LST programmes should proceed once key enablers such as information systems, governance, and engagement/involvement structures are in place. Collaboratively setting and refreshing a clear, locally contextualised vision, and creating a sense of ownership among stakeholders, are also essential [34].

Local Maternity Systems in England, along with the ICSs and STPs in which they are located, are evolving, and as yet have no statutory authority, and it has been suggested that legislative change is necessary [35]. The boundaries between different organizations and networks are often blurred, meaning that leaders cannot rely solely on formal authority to influence change [36]. In our study reorganisations were successful in integrating some structures, facilitated by a programme team, however, there were limits to what could be achieved where accountability was to individual organisations and not the system. This disconnect was a significant barrier to LST because, as Turner et al. highlighted in their development of Best et al’s rules, LST is unlikely in the absence of system-wide authority [24]. Leaders were also expected to establish the system in a context of organisational competition, rather than collaboration. It has been suggested that delegating authority to a third party may be the best solution in such situations [37]. Local power dynamics made the need to ensure parity of representation and involvement in the LST at every level evident, which was only recognised part-way into the programme.

This LST was a more complex undertaking than organisation-level change which has been the focus of training and experience of most NHS leaders in recent years, though this situation is changing [38]. Systems thinking and practice has its own underlying theory and evidence-base, which did not feature prominently in the LST we studied. While some participants reported a move towards a systems perspective over time, our findings confirm those of others that suggest further efforts are required to embed it alongside more familiar organisational change practices [39]. Experience of past NHS change programmes offer limited insight into how to structure a system-level LST, which necessitated learning and adaptation over time. The initial structure, informed by the national Maternity Transformation Programme, proved to be complex, fragmented, and challenging to deliver with available capacity, and was consolidated, as observed in LST elsewhere [40]. As the programme developed the importance of balancing leadership and involvement across organisations, and connecting the LST work and structures with core business and hierarchies of providers, were increasingly apparent. Restructuring in response to this learning, while welcomed, inevitably led to discontinuity in leadership, and disruption in the LST, with stability a key feature of successful transformation [41].

### The policy dimension

The national policy driving the LST facilitated change in maternity care, which has historically been perceived as low priority compared with other areas of health [42]. The national programme raised the profile of maternity among senior leaders, leveraged funding, and provided the opportunity to build a network of LST leaders to share learning across the country.

The larger and more complex the LST, the greater the difficulty of its implementation [43]. A flexible and iterative approach is necessary to bring about complex change [44], and while localities were able to develop the approach to implementing *Better Births*, the centrally fixed policy objectives hampered progress. In terms of feedback loops, nationally set measures of ‘success’ led to the focus of effort in specific areas (such as continuity of carer) at the expense of others (maternity hubs), a recognised risk of LST [45], and some measures became more stringent as policy was refined over time. Some recommendations and targets were dealt with separately, rather than as interdependent aspects of system change. This was exacerbated by the capacity and expertise of stakeholders to lead integrated system change of this scope and scale, and a complex LST structure. The specific area focus may have been mitigated by setting targets which encompass policy recommendations more equally (e.g. NHS targets did not focus on community maternity hubs), and which fostered integration across policy areas (e.g. by setting targets requiring continuity of carer teams to also be based in community hubs). Integration of the implementation of the recommendations could have been further enhanced by drawing on and communicating relevant evidence as part of a compelling vision for the programme; for example, utilising evidence that continuity of carer models improve choice, personalisation, and reduce inequalities [46]. While a wide range of stakeholders was involved in *Better Births*, staff in our study frequently questioned continuity of care policy and targets, which affected their delivery. Implementation may have been facilitated by more collaboration with midwives in specifying targets [47]. Work continues to encourage staff engagement and adoption of continuity [48].

Consistent with Best et al’s rules about involving patients and engaging stakeholders, women and professionals were involved in the development of the national policy. Policymakers also expected local areas to involve women, although the MVPs, the mechanisms for achieving this, were not launched until the LST was in progress, and while some maternity systems in England seamlessly developed their existing structures (MSLCs) into MVPs, in our study site a fresh approach was adopted, creating temporary discontinuity in women’s involvement. Stakeholder perspectives can vary, a common challenge when seeking an inclusive approach to LST [30, 49, 50]. We found women and some staff shared different priorities, for instance a desire to reduce maternity health inequalities. Inequalities were not addressed directly in the *Better Births* recommendations, whereas *Best Start*, the Scottish maternity review, while broadly similar, included specific recommendations regarding the needs of women with vulnerabilities [51]. Maternity health inequalities have since been identified as a national priority in England [52]. Divergence had a negative impact on engagement and involvement as women and staff felt their views were overlooked. While there was scope to pilot new ways of working, there was an implicit expectation at national policy level that they would work from the outset [6]. Leaders’ ability to test and challenge policy was restricted, because their performance would be judged on policy success as defined centrally [53]. The ability to fully involve and engage staff and the public in policy implementation was curtailed by a focus on ‘selling’ the change [6], and communicating ‘good news’ [54]. Recent work by Harris *et al* has highlighted continued resistance of many midwives at national level to continuity of care policy in particular, and the complex influences on willingness and perceived ability to adopt continuity models [48]. Their development and national evaluation of a midwifery workshop intervention, drawing on behaviour change theory, identified that barriers are amenable to change, but that organisation and management need to play a substantial role in overcoming them. They conclude that further work is needed to develop effective change interventions and influencing and midwife perceptions is necessary if policy aims are to be achieved [47].

### Interdependence of the rules for LST

Our analysis builds on the five rules framework, theorising how adherence to one enhances how others are followed, suggesting that they must be viewed as interdependent and synergistic, rather than separate approaches to successful LST, aligning with broader complexity science and systems thinking [55]. The influences on adherence to rules are consistent with the evidence base regarding facilitators and constraints to change programmes in healthcare, such as capacity, funding, and organisational culture [56, 57].

### Strengths and limitations

This in-depth, longitudinal study of change traced a programme of LST over two years. It captured events and experiences as they happened. Diverse sources and participants provided extensive and rich data which enabled triangulation to provide a more complete account of the LST, beyond a limited focus on central leaders [58]. The systematic approach to analysis, involving three researchers working together closely and iteratively, enhanced rigour. Analysis included interrogation of data to explore divergent cases and themes: though none were identified, we identified groups of individuals with divergent perspectives as discussed in the findings section, for example mismatched MVP and NHS stakeholder expectations of involvement work. Interpretation was strengthened by reflecting on findings with the programme leaders [59]. However, a limitation of the project was that we were unable to include national and regional policymakers as participants.

The Best et al. rules were developed from a realist synthesis, providing an evidence-based framework to understand ‘what works’ drawing on many contexts. Recent work has refined the rules further, and we incorporated this into our analysis [24]. Compared with other frameworks, the relative simplicity of the rules facilitated coding and analysis. The rules were relatively straightforward to communicate to external audiences, including with leaders during the evaluation.

The simplicity of Best et al’s rules also has limitations. Reducing ‘what works’ to five rules does not account for the variety, complexity and scale of LST, rests on the assumption that LST is needed [6], and does not account for some of the individual actions and perspectives [23]. Any simple framework, whilst potentially helpful in identifying broad principles for LST, cannot fully account for the granular nature of change in action. The rules are also open to interpretation. For example, we found contrasting stakeholder perspectives of what ‘engagement’ meant [54].

While such frameworks abound in the literature on health system change, their translation to practice is often problematic. The Best et al. rules provide a lens through which to explore LST efforts, but they also provide a way of ‘not seeing’ change. Leaders were provided with the rules, and they were used as a framework to present interim findings, but we found it revealing that they were not embraced by the people confronted with implementing change, with a number of possible explanations. The rules present a broad set of principles rather than a ‘how to’ guide for transformation. While Best et al’s work is highly cited in the academic literature, we have yet to find any reference to it in guidance for NHS leaders, reflecting the disconnect between academic and in-practice fields [60].

## Conclusion

Any programme of LST needs to have realistic objectives, with timescales that account accurately for both resources and constraints. In the course of implementing change in healthcare, such programmes also need to be sufficiently flexible to adapt to feedback from what are inherently complex systems. Our case study found that pace, scale and complexity meant expectations about what could be achieved, and how rapidly, were not met in full. Here, a national transformation programme provided the resources and impetus for change but – as is the case with other public services - it is extremely challenging to implement LST against the backdrop of continual reforms.

The rules, as we have synthesised them to apply to a health services research perspective, may not account for all that is important. For example, a frequent assumption in LST is that the changes are wanted and necessary [6]. For this LST, divergent expectations and interests meant it was widely experienced as a politically-driven project. At the same time as lacking an appreciation for this kind of *realpolitik*, the Best rules are freighted with a kind of idealism. They presume change will occur, whereas the history of healthcare reforms teaches us there may be little evidence to confirm this. As well as being prepared to account for change, decision makers themselves have to be pragmatists. They stand ready to temper expectations about the true scale of change, and are even open to asking whether change is possible [61].

## Data Availability

The datasets generated and analysed during the current study are not publicly available as consent was not provided by participants to share their data with third parties.

## Abbreviations

LST: Large System Transformation
ICS: Integrated Care System
STP: Sustainability and Transformation Partnership
NHS: National Health Service
LMS: Local Maternity System
MVP: Maternity Voices Partnership
